# A case of gastric and duodenal mucosa-associated lymphoid tissue lymphoma with multiple gastric cancers: a case report

**DOI:** 10.1186/s40792-020-01081-8

**Published:** 2021-01-25

**Authors:** Takashi Yokoyama, Tetsuya Tanaka, Suzuka Harada, Takeshi Ueda, Goki Ejiri, Shoh Sasaki, Maiko Takeda, Atsushi Yoshimura

**Affiliations:** 1 Department of Surgery, Minami-Nara General Medical Center, 8-1 Fukugami, Oyodo, Yoshino, Nara 638-8551 Japan; 2 Department of Pathology, Nara Medical University Hospital, 840 Shijo-cho, Kashihara, Nara 634-8521 Japan

**Keywords:** Gastric MALT lymphoma, Duodenal MALT lymphoma, Gastric cancer, Multiple cancers

## Abstract

**Background:**

Gastric mucosa-associated lymphoid tissue (MALT) lymphoma is often caused by *Helicobacter pylori* and has a good prognosis. Rarely, patients with MALT lymphoma may have gastric cancer and have a poor prognosis.

**Case presentation:**

We herein report a case in which surgical treatment was achieved for a 72-year-old male patient with gastric and duodenal MALT lymphoma coexisting multiple gastric cancers. He underwent upper endoscopy for epigastric discomfort, which revealed mucosal erosion on the posterior wall of the middle body of the stomach, an elevated lesion on the duodenal bulb, and a raised tumor on the antrum of the stomach. He was diagnosed with gastric and duodenal MALT lymphoma with early gastric cancer. One month after *H. pylori* eradication, a second upper endoscopy revealed no improvement in the gastric or duodenal mucosa, and areas of strong redness with a shallow recess just below the cardia of the stomach. As a result, a diagnosis of gastric and duodenal MALT lymphoma with two gastric cancers was made. Total gastrectomy with proximal duodenum resection using intraoperative upper endoscopy and regional lymph node dissection was performed. Pathologically, gastric and duodenal MALT lymphoma and three gastric cancers were detected. Since one of them was an advanced cancer, he started taking S-1 after his general condition improved.

**Conclusion:**

For early detection of gastric and duodenal MALT lymphoma or gastric cancer, appropriate upper endoscopy and a biopsy are important. It is necessary to select a suitable treatment, such as *H. pylori* eradication, endoscopic treatment, surgery, chemotherapy, and irradiation, according to the disease state.

## Background

Mucosa-associated lymphoid tissue (MALT) lymphoma is a low-grade lymphoma derived from marginal zone B cells of MALT that occurs in extranodal organs, such as the digestive tract, salivary gland, thyroid gland, lung, bladder, and skin. The disease concept of MALT lymphoma was first proposed by Issacson et al. in 1983 [1]. Among the gastrointestinal MALT lymphomas, the primary site is the stomach (60–70%), followed by the small intestine (20–30%) and large intestine (5–15%), with the esophagus being quite rare (< 1%) [2]. The simultaneous coexistence of an adenocarcinoma associated with a gastric MALT lymphoma is a rare entity but is well known. The first-line treatment for MALT lymphoma and gastric cancer is, respectively, eradication of *Helicobacter pylori* and endoscopic or surgical resection, but the treatment for coexisting cases has been controversial.

We herein report a case of gastric and duodenal MALT lymphoma coexisting with multiple gastric cancers and focus on the problems often encountered in such rare instances.

## Case presentation

**Fig. 1:**
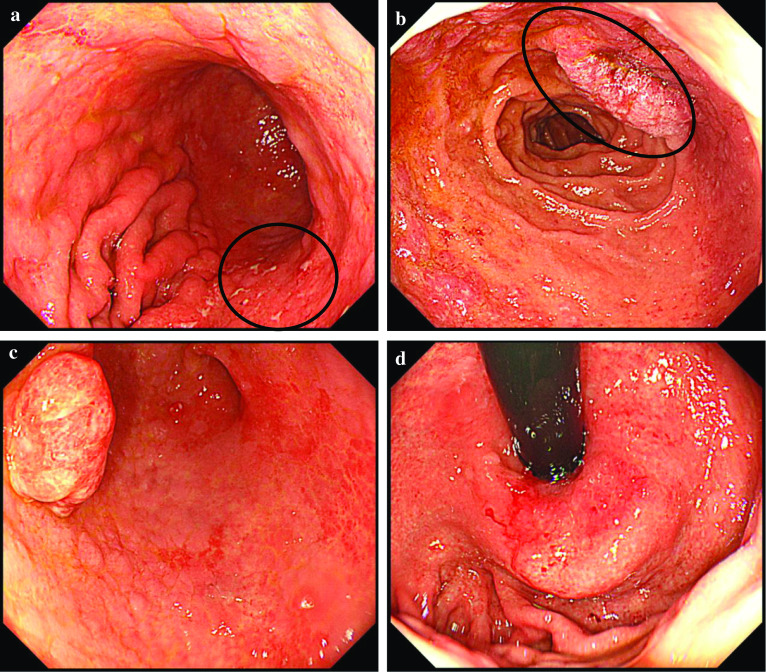
Upper gastrointestinal endoscopic findings. **a** Mucosal erosion (circle) on the posterior wall of the middle body of the stomach. **b** Elevated lesion (circle) on the duodenal bulb. **c** Raised tumor on the antrum of the stomach. **d** Strong redness with shallow recess just below the cardia

A 72-year-old man underwent upper endoscopy for epigastric discomfort, which revealed mucosal erosion on the posterior wall of the middle body of the stomach (Fig. 1a), an elevated lesion on the duodenal bulb (Fig. 1b), and a raised tumor on the antrum of the stomach (Fig. 1c). A histopathologic examination of the mucosal erosion on the posterior wall of the middle body of the stomach and elevated lesion on the duodenal bulb showed that small atypical lymphoid cells proliferated diffusely and occasional lymphoepithelial lesions were also present. Immunohistochemically, the lymphoid cells were positive for CD20 and BCL2, while they were negative for CD10. Moderately differentiated adenocarcinoma was revealed in the antrum of the stomach. Abdominal computed tomography (CT) showed a tumor 2 cm in size in the antrum of the stomach and no signs of lymph node or liver metastasis. Based on these examinations, the initial diagnosis was gastric and duodenal MALT lymphoma with early gastric cancer. The urea breath test showed *H. pylori* infection. To avoid postoperative MALT lymphoma remnants and shorten the resection range of the duodenum, *H. pylori* eradication therapy, which was expected to reduce gastric and duodenal MALT lymphoma lesions, was thus performed.

**Fig. 2:**
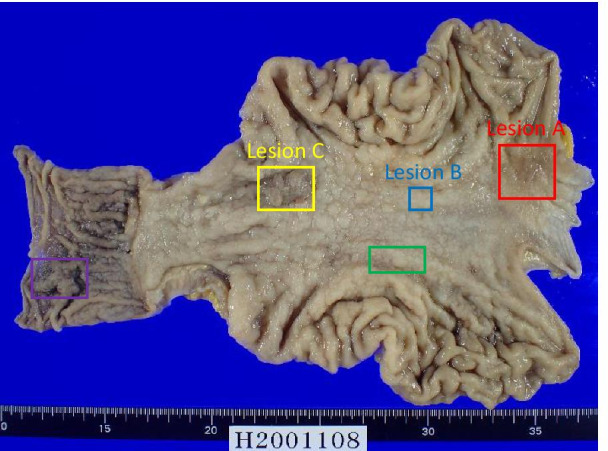
The resected specimen of the stomach and proximal duodenum. The red, blue or yellow frames show adenocarcinomas in the lesion A, B, or C, respectively. The green or purple frames indicate MALT lymphomas in the stomach or duodenum, respectively

**Fig. 3:**
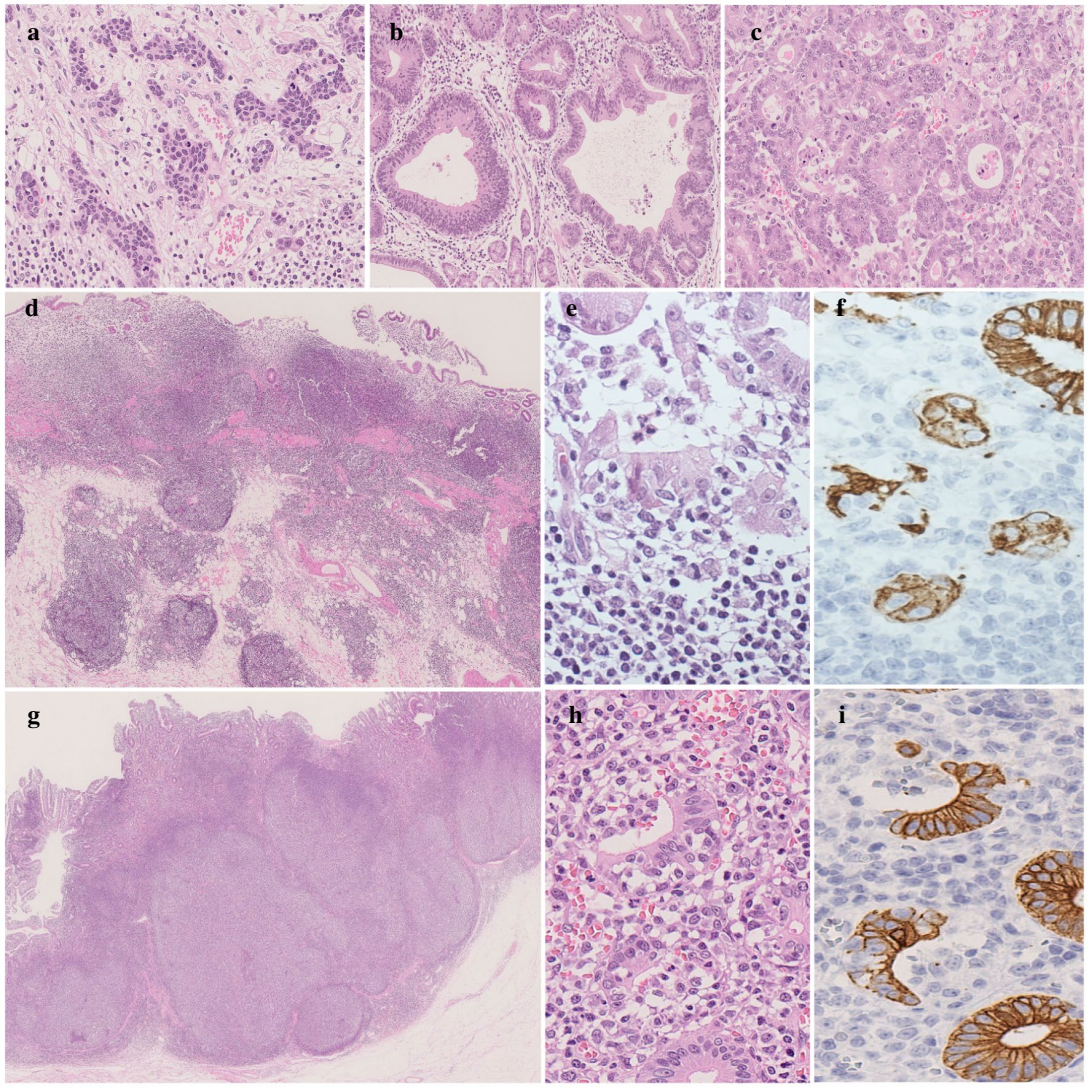
Microscopic pathological findings. **e**, **f** or **h**, **i** are serial sections, respectively. **a** Poorly differentiated adenocarcinoma, non-solid type in the lesion A. **b** Tubular adenocarcinoma, well differentiated in the lesion B. **c** Tubular adenocarcinoma, moderately differentiated in the lesion C. **d**–**i** MALT lymphoma in the **d**–**f** stomach or **g**–**i** duodenum indicated by the green or purple frame in **Fig. 3**. MALT lymphoma cells diffusely proliferate with (**e**, **h**) lymphoepithelial lesions highlighted by **f**, **i** CAM5.2. Original magnification, **a**–**c**, **e**–**f**, **h**, **i**: ×400; **d**, **g**: ×20

One month after performing eradication therapy, the second upper endoscopy revealed no improvement in the gastric or duodenal mucosa, and an area of strong redness with a shallow recess just below the cardia of the stomach (Fig. 1d). Poorly differentiated adenocarcinoma was revealed in the cardia of the stomach. The preoperative diagnosis was gastric and duodenal MALT lymphoma with two gastric cancers. One month after the last upper endoscopy, total gastrectomy with proximal duodenum resection using intraoperative upper endoscopy and regional lymph node dissection was performed. No improvement in either the gastric or duodenal mucosa was observed by intraoperative endoscopy. Three cancer lesions found in whole tissue sections of a resected specimen after fixation were examined according to the 15th edition of Japanese Classification of Gastric carcinoma (Fig. 2). In lesion A, measuring 3 × 3 cm and located in the cardia, cancer cells arranged in small nests and cords had proliferated and invaded the subserosal layer (por2, T3) (Fig. 3a). In lesion B, measuring 1 × 1 cm and located in the small curvature of the middle corpus, cancer cells arranged in well-formed glands had proliferated and remained in the lamina propria (tub1, T1a) (Fig. 3b). In lesion C, measuring 2.5 × 1.5 cm and located in the small curvature of the antrum, cancer cells arranged in fused glands had proliferated and remained in the lamina propria (tub2, T1a) (Fig. 3c). Of the 60 perigastric lymph nodes, 1 showed metastatic carcinoma (N1). In both the posterior wall of the middle body of the stomach (Fig. 3d, e) and the duodenal bulb (Fig. 3g, h), small atypical lymphoid cells proliferated diffusely and deeply from the lamina propria to the submucosa, and occasional lymphoepithelial lesions were present. Immunohistochemically, the lymphoid cells were positive for CD20 and BCL2 and negative for CD10, and lymphoepithelial lesions were highlighted by CAM5.2 (Fig. 3f, i). The morphological features and immunohistochemical staining patterns supported the diagnosis of gastric and duodenal MALT lymphoma. No MALT lymphoma lesions were detected outside the range of green and purple frames in Fig. 2.

We diagnosed the disease as stage IIB (T3N1M0) gastric cancer (the 15th edition of Japanese Classification of Gastric carcinoma) and stage I gastric and duodenal MALT lymphoma (Lugano classification). The patient was discharged 11 days after surgery and received adjuvant chemotherapy (S-1 100 mg/day/body).

## Discussion

Non-Hodgkin lymphoma (NHL) is a common hematologic malignancy worldwide. The gastrointestinal tract is the most common extranodal primary site for NHL, accounting for 40% of all primary extranodal lymphomas. Gastrointestinal lymphomas include several histologic subtypes: MALT lymphoma, diffuse large B-cell lymphoma, Burkitt lymphoma, enteropathy-associated T-cell lymphoma, mantle cell lymphoma, and follicular lymphoma. MALT lymphoma accounts for 40% of gastrointestinal lymphomas [2].

In 1983, Isaacson and Wright first reported the concept of MALT lymphoma, which includes low-grade B-cell lymphoma and immunoproliferative disease [1]. Primary gastrointestinal MALT lymphoma can involve any part of the gastrointestinal tract, from the esophagus to the rectum. The most involved site of MALT is the stomach (60–70%), followed by the small intestine, ileum, cecum, and rectum [2]. Gastric MALT lymphoma usually affects older patients and is evenly distributed between men and women. Symptoms often resemble those of gastritis and peptic ulcer, including upper abdominal discomfort and pain, nausea and vomiting, and rarely bleeding and weight loss [3]. Regarding duodenal MALT lymphoma, little is known about the etiology, pathogenesis presenting manifestations, or treatment due to its rarity.

Several cases of coexisting gastric cancer and MALT lymphoma have been reported [4–7]. However, while the rate of such coexistence is high, gastric cancer lesions are easily misdiagnosed as multifocal separated MALT lymphoma lesions because of their similar endoscopic findings. In our case, two gastric cancers (lesion A, C) were noted on preoperative upper endoscopy, but one (lesion B) was only able to be detected pathologically. Therefore, patients with gastric MALT lymphoma should be carefully examined by endoscopy, and suspicious areas should be biopsied for the possible existence of adenocarcinoma. Such coexistence is divided into three types: separated type, collision type, and mixed type, and the frequency is high in this order. In the present case, cancer and MALT lymphoma were the separated type, showing no mixing and not in contact with each other on a pathological examination in whole tissue sections of a resected specimen. Gastric malignant lymphoma is hypothesized to cause gastric cancer, since gastric malignant lymphoma, including MALT lymphoma, is frequently associated with gastric cancer [8]. In the separated type, gastric cancer and MALT lymphoma are not in contact with each other; they are thus considered to develop with *H. pylori* as a common tumor factor. Uemura et al. performed a cohort study of endoscopic surveillance of gastric cancer and found that all occurrences of gastric cancer in the cohort were in *H. pylori*-infected subjects [9]. Given these findings, *H. pylori* infection was included in the previously proposed gastric carcinogenesis process, known as Correa’s cascade [10].

Wotherspoon et al. reported that *H. pylori* infection was present in 92% of patients with MALT lymphoma [11]. The exact mechanism underlying the transition from *H. pylori* infection to MALT lymphoma is still unclear. The most promising hypothesis is that a chronic immunological antigen stimulation by *H. pylori* increases lymphoid tissue, and microenvironmental factors and genetic predisposition are subsequently added to the lymphoid tissue, leading to tumorigenesis [12, 13].

For gastric MALT lymphoma, the ideal treatment option is the eradication of *H. pylori*, which results in complete remission in approximately 80% of all patients [14, 15]. Remission seems to be maintained in most cases for years. Gastric MALT lymphoma is currently the only cancer that can be treated by a simple antibiotic treatment. Eradication of *H. pylori* directly induces apoptosis in inflammation-related immunocytes in the gastric mucosa, and this may be the mechanism underlying the cure of gastric MALT lymphoma. In duodenal MALT lymphoma, the role of *H. pylori* is not clear, but Nagashima et al. reported the regression of duodenal MALT lymphoma following the eradication of *H. pylori* [16]. In the present case, eradication therapy of *H. pylori* was administered initially, but no improvement in the gastric or duodenal mucosa was observed. *H. pylori* negative, t (11; 18)/API2-MALT1 positive, B-cell monoclonality by PCR, localization in the upper stomach, and deep submucosal infiltration of the stomach wall have been reported as resistance factors for *H. pylori* eradication therapy [14][14][14]. For gastric MALT lymphoma patients not responding to *H. pylori* eradication, the strategy is still controversial. The “watch and wait” strategy is also recommended for patients with persistent histologic lymphoma without progressive disease [17]. For patients demonstrating progressive disease with involvement of the distant lymph nodes and/or bone marrow, it was reported that 95% of MALT lymphoma patients who failed to successfully undergo *H. pylori* eradication showed complete remission after combined chlorambucil and rituximab therapy [19]. Total gastrectomy is required to ascertain the pathology and staging of the lymphoma, but in recent years, surgery has played a less important role in treating gastric MALT lymphoma. A meta-analysis of 5 studies with 700 patients showed an equal overall survival and a better disease-free survival with medication (chemotherapy and/or radiotherapy) than surgery (surgery alone or combined with chemotherapy and/or radiotherapy), and the mortality was higher in the surgery group than in the medication group [20]. As lymphoma is a systemic disease, a systemic approach like chemotherapy is appropriate. Surgery should be restricted to the treatment of coexisting cancer and complications, such as bleeding or perforation.

For gastric cancer, it is undisputed that complete resection, such as surgery or endoscopic resection, is the first-line treatment for a radical cure. Gastric MALT lymphoma patients with early or advanced gastric cancer can be cured by the eradication of *H. pylori* and endoscopic treatment or surgical procedures. For patients with multiple gastric cancers, total gastrectomy should be performed, as patients with multiple gastric cancers who undergo partial resection reportedly have a higher frequency of residual gastric cancer than those with single gastric cancer [21]. Furthermore, adjuvant chemotherapy with S-1 has been recommended to improve the survival in patients with stage II/III gastric cancer who underwent curative resection [22].

## Conclusion

Gastric MALT lymphoma is a relatively rare disease and does not show specific symptoms or endoscopic findings, so the early diagnosis is difficult. However, it is also known that gastric cancer sometimes coexists in patients with MALT lymphoma. A careful endoscopic examination is necessary for the early detection of these diseases. Regarding the therapy, it is therefore necessary to carefully consider *H. pylori* eradication, endoscopic treatment, surgery, chemotherapy, or irradiation depending on the spread, site, and stage of MALT lymphoma or gastric cancer.

## Data Availability

All data supporting this article are included in the published article.

## Abbreviations

MALT: Mucosa-associated lymphoid tissue
*H. pylori*: *Helicobacter pylori*
CT: Computed tomography
NHL: Non-Hodgkin lymphoma
